# TRAFICA: an open chromatin language model to improve transcription factor binding affinity prediction

**DOI:** 10.1093/bioinformatics/btaf469

**Published:** 2025-08-23

**Authors:** Yu Xu, Chonghao Wang, Ke Xu, Yi Ding, Aiping Lyu, Lu Zhang

**Affiliations:** Department of Computer Science, Hong Kong Baptist University, 999077 Hong Kong, China; Department of Computer Science, Hong Kong Baptist University, 999077 Hong Kong, China; Department of Computer Science, Hong Kong Baptist University, 999077 Hong Kong, China; Department of Computer Science, Hong Kong Baptist University, 999077 Hong Kong, China; School of Chinese Medicine, Hong Kong Baptist University, 999077 Hong Kong, China; Institute of Systems Medicine and Health Sciences, Hong Kong Baptist University, 999077 Hong Kong, China; Department of Computer Science, Hong Kong Baptist University, 999077 Hong Kong, China; Institute of Systems Medicine and Health Sciences, Hong Kong Baptist University, 999077 Hong Kong, China

## Abstract

**Motivation:**

*In silico* transcription factor and DNA (TF–DNA) binding affinity prediction plays a vital role in examining TF binding preferences and understanding gene regulation. The existing tools employ TF–DNA binding profiles from *in vitro* high-throughput technologies to predict TF–DNA binding affinity. However, TFs tend to bind to sequences in open chromatin regions *in vivo*, such TF binding preference is seldomly considered by these existing tools.

**Results:**

In this study, we developed TRAFICA, an open chromatin language model to predict TF–DNA binding affinity by integrating sequence characteristics of open chromatin regions from ATAC-seq experiments and *in vitro* TF–DNA binding profiles from high-throughput technologies. We pretrained TRAFICA on over 2.8 million nucleotide sequences in open chromatin regions derived from 197 ATAC-seq experiments (115 cell lines) to learn *in vivo* TF binding preferences. We further fine-tuned TRAFICA using low-rank adaptation (LoRA) on PBM and HT-SELEX TF-DNA binding profiles to learn intrinsic binding preferences for specific TFs. We systematically evaluated TRAFICA and compared its predictive performance with existing prediction tools and advanced DNA language models. The experimental results demonstrated that TRAFICA significantly outperformed the others in predicting *in vitro* and *in vivo* TF–DNA binding affinity, achieving state-of-the-art performance. These findings indicate that considering the sequence characteristics from open chromatin regions could significantly improve TF–DNA binding affinity prediction.

**Availability and implementation:**

The source code of TRAFICA and detailed tutorials are available at https://github.com/ericcombiolab/TRAFICA.

## 1 Introduction

The binding of transcription factors (TFs) to DNA is a crucial step in gene regulation (Oli *et al.* 2021, Loupe *et al.* 2024), where TFs recognize and interact with specific promoter sequences in genomes to regulate the expression of nearby downstream genes. The *in vitro* TF–DNA binding affinity is primarily attributed to intrinsic TF binding preferences, which can be captured through *in vitro* high-throughput experiments in the absence of cellular environments (Orenstein and Shamir 2014). The *in vivo* TF–DNA binding affinity is more intricate, because it not only relates to intrinsic TF binding preferences but is also influenced by other factors, such as the positions of nucleosomes and the cobinding mechanism (Slattery *et al.* 2011, Lambert *et al.* 2018). Nucleosome positions can restrict the accessibility of TF binding sites, thus TFs could only bind to DNA sequences in open chromatin regions; the cobinding mechanism refers to the cooperative binding of multiple TFs and cofactors, such as the transcriptional corepressor TUP1–SSN6 complex in yeast (Johnson 1995).

**Figure 1.**
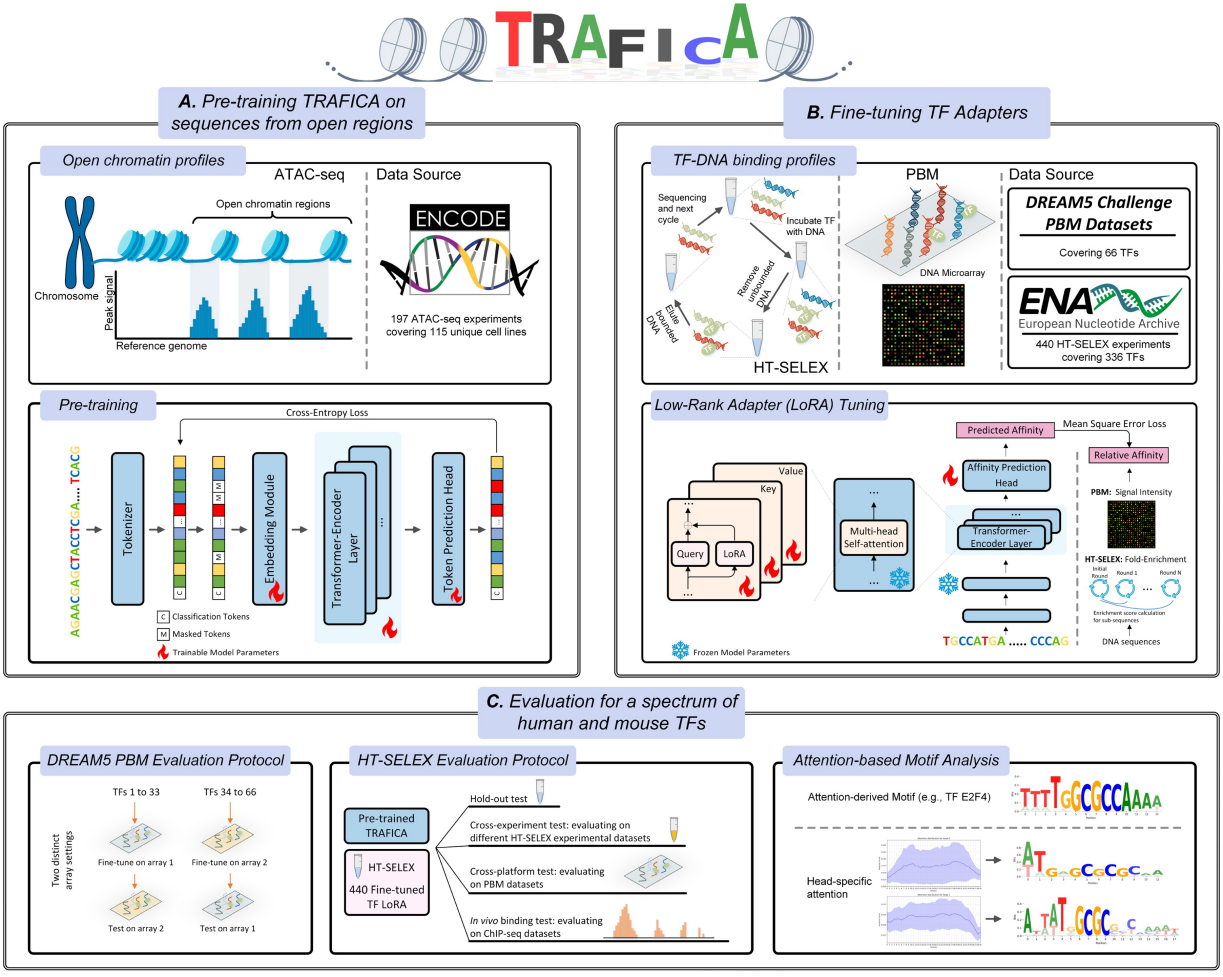
Overview of TRAFICA. (A) Pretraining stage. TRAFICA was pretrained on ATAC-seq profiles from 115 ENCODE cell lines using masked token prediction to learn open chromatin context. The constructed ATAC-seq dataset comprised 2.8 million accessible genomic regions. (B) Fine-tuning stage. TRAFICA was fine-tuned on DREAM5 PBM and ENA HT-SELEX datasets to learn binding preferences for specific TFs. LoRA was applied exclusively to query, key, and value projection layers in multihead self-attention modules. (C) Systematic evaluation. Two evaluation paradigms were used: (i) the established DREAM5 PBM evaluation protocol and (ii) our newly developed HT-SELEX evaluation protocol. Additionally, we performed attention score analysis to interpret the biological relevance of TRAFICA, and derived TF binding motifs.

Several technologies have been developed to measure TF binding preferences, both *in vivo* and *in vitro*. Chromatin ImmunoPrecipitation followed by sequencing (ChIP-seq) (Johnson *et al.* 2007) is a popular *in vivo* sequencing technology to collect the binding sequences of a specific TF across the whole genome. ChIP-seq has a significant limitation, as the antibodies for chromatin immunoprecipitation may not always be available for many TFs (Lambert *et al.* 2018). Protein Binding Microarray (PBM) (Berger *et al.* 2006) is an *in vitro* high-throughput technology that measures the binding affinities of a specific protein to a set of artificial DNA sequences according to fluorescence intensities. High-Throughput Systematic Evolution of Ligands by EXponential enrichment (HT-SELEX) (Jolma *et al.* 2010) is a well-established *in vitro* technology, which involves multicycle selection to screen sequences binding to a target protein. This technology could yield abundant sequences with high affinity to a TF of interest. Moreover, Assay for Transposase-Accessible Chromatin using sequencing (ATAC-seq) (Buenrostro *et al.* 2015) is designed to capture chromatin accessibility (Fig. 1A), which is a key factor that could influence *in vivo* TF binding. The intrinsic preferred sequences (e.g. promoter and enhancer sequences) of TFs are also typically found in open chromatin regions (Boyle *et al.* 2008). The rapid development of these technologies has contributed to large-scale publicly available datasets that have yet to be fully utilized (Leinonen *et al.* 2011, The ENCODE Project Consortium 2012, Luo *et al.* 2020).

In recent years, there has been a growing interest in developing tools based on classic machine learning models (Alipanahi *et al.* 2015, Li *et al.* 2017, Wang *et al.* 2021, Zhang *et al.* 2021, Barissi *et al.* 2022) to predict TF–DNA binding affinity using TF–DNA binding profiles generated by PBM and HT-SELEX experiments. DNAshapeR (Li *et al.* 2017) employs a multiple linear regression model with *L*2 penalty and utilizes 13 DNA shape features (including interbase pair types, intrabase pair types, and minor groove width) from DNA sequences to predict TF-DNA binding affinity. DNAffinity (Barissi *et al.* 2022) represents a sequence as a feature vector composed of four classes of DNA properties [base pair (bp) parameters, stiffness, sequence pattern, and electrostatics] and employs a random forest regression model for TF–DNA binding affinity prediction. However, classic machine learning-based tools typically require feature engineering on DNA sequences to obtain fixed-length features to describe the characteristics of nucleotide sequences. In contrast, deep learning-based models could directly learn characteristics from sequences with different lengths instead of relying on feature engineering. DeepBind (Alipanahi *et al.* 2015) applies convolutional neural networks (CNNs) to capture binding motifs by CNN kernels and predict TF binding preferences. DLBSS (Zhang *et al.* 2021) integrates four DNA shape features as inputs of CNNs for TF-DNA binding affinity inference. CRPTS (Wang *et al.* 2021) combines a long short-term memory network with CNNs as a hybrid model to improve predictive performance. Although the existing tools have demonstrated advancements in predicting *in vitro* TF–DNA binding affinity, they have yet to consider the characteristics of DNA sequences from open chromatin regions. Moreover, Transformer-based DNA language models including DNABERT (Ji *et al.* 2021), DNABERT2 (Zhou *et al.* 2023), and Nucleotide Transformer (Dalla-Torre *et al.* 2025) have significantly advanced nucleotide sequence analysis, which are typically pretrained on the whole reference genome.

In this study, we developed TRAFICA (TRanscription factor binding AFfinity prediction Integrating Chromatin Accessibility), an open chromatin language model that integrates the characteristics of DNA sequences from open chromatin regions and *in vitro* TF–DNA binding profiles from high-throughput technologies to predict TF–DNA binding affinity. TRAFICA is built on the Transformer-encoder architecture (Vaswani *et al.* 2017) with Rotary Position Embedding (RoPE) (Su *et al.* 2024), which includes a pretraining stage to capture sequence contextual characteristics based on over 2.8 million genomic accessible sequences from ATAC-seq experiments (Fig. 1A). The pretrained model was fine-tuned on known TF-DNA binding profiles using low-rank adapter (LoRA) (Hu *et al.* 2022) to learn specific TF binding preferences (Fig. 1B). We evaluated TRAFICA in two paradigms (Fig. 1C): (i) employing the DREAM5 challenge PBM datasets to benchmark TRAFICA against existing tools, including classic machine learning-based [DNAshapeR (Li *et al.* 2017) and DNAffinity (Barissi *et al.* 2022)] and deep learning-based [DeepBind (Alipanahi *et al.* 2015), DLBSS (Zhang *et al.* 2021), and CRPT/CRPTS (Wang *et al.* 2021)] tools, as well as pretrained DNA language models [DNABERT (Ji *et al.* 2021), DNABERT2 (Zhou *et al.* 2024), and Nucleotide Transformer (Dalla-Torre *et al.* 2025)] and (ii) systematically assessing HT-SELEX fine-tuned models across multiple test sets (HT-SELEX, PBM, and ChIP-seq) compared to DNA language models (see Section 2). Additionally, we evaluated the impact of tokenization approaches for DNA sequences on TF–DNA binding prediction, including *k*-mer (*k *= 4, 5, and 6; single-base: *k *= 1) and byte-pair encoding (BPE) (Sennrich *et al.* 2016) tokenizations. TRAFICA consistently outperformed all competing tools across the evaluations, demonstrating its robustness as a computational tool for investigating TF–DNA binding.

## 2 Materials and methods

### 2.1 HT-SELEX evaluation protocol

We applied a binning technique to a target HT-SELEX dataset to group sequences based on their binding affinities, resulting in $N_{\text{sub}}$ bins. Next, we randomly selected a sequence from each bin to construct a subset with $N_{\mathrm{sub}}$ sequences. More specifically, denoted $v_{\text{max}}$ ($v_{\text{min}}$) as the maximum (minimum) affinity value, we calculated the range of bins as $v_{\text{bin}}=(v_{\text{max}}-v_{\text{min}})/N_{\text{sub}}$. Thus, the edges of bins are {$\left[v_{\text{min}},v_{\text{min}}+v_{\text{bin}}\right)$, $\left[v_{\text{min}}+v_{\text{bin}},v_{\text{min}}+2\times v_{\text{bin}}\right)$, …, $\left[v_{\text{min}}+(N_{\text{sub}}-1)\times v_{\text{bin}},v_{\text{max}}\right]$}. This sampling procedure was repeated three times without replacement, resulting in dataset splits of *N* (fine-tuning), N/10 (validation), and N/10 (hold-out test) sequences (N∈{10 000,20 000,30 000,40 000,50 000}), respectively. In addition to hold-out testing, we evaluated model generalizability by applying fine-tuned models to predict binding affinity on independent HT-SELEX (cross-experiment test) and PBM (cross-platform test) datasets. Moreover, we collected cell line ChIP-seq profiles from the ENCODE project to construct *in vivo* test sets. More details of ChIP-seq dataset construction can be found in Note 1, available as supplementary data at *Bioinformatics* online.

### 2.2 Nucleotide sequence tokenization

We processed each nucleotide sequence into an ordered list of tokens using several tokenization approaches, including single-base, *k*-mer (*k *= 4, 5, and 6), and BPE tokenizations. For *k*-mer tokenization, each token is represented by *k* consecutive nucleotides. We constructed a vocabulary of *k*-mer tokens comprising all possible *k*-mers with four symbols (“A,” “T,” “G,” and “C”) to represent DNA nucleotides. For BPE tokenization, we constructed a custom vocabulary using SentencePiece (Kudo and Richardson 2018) on our ATAC-seq pretraining corpus. To enable direct comparison with DNABERT2, we also pretrained a TRAFICA version using the BPE vocabulary developed in DNABERT2. Following previous studies (Devlin *et al.* 2018, Ji *et al.* 2021), we also introduced five special tokens into all vocabulary configurations, including “[CLS],” “[SEP],” “[MASK],” “[PAD],” and “[UNK].” This yields vocabulary sizes of: (single-base) 4+5, (*k*-mer) $4^{k}+5$, and (BPE) 4096+5.

### 2.3 Model pretraining

During the pretraining stage, we randomly masked tokens for the versions of single-base and BPE tokenizations to perform self-supervised learning (Fig. 1A). For *k*-mer tokenization, masking single tokens could oversimplify the model pretraining, as they could be completely recovered by their preceding and succeeding tokens (Ji *et al.* 2021). For example, in a tokenized sequence “AGAA [MASK] AACG,” the masked token can be ascertained as “GAAC” by its neighbor tokens. To address this issue, we masked $l_{\text{mask}}$ (default: 10) consecutive tokens (e.g. “AGAA [MASK] … [MASK] [MASK] CGTA”) instead of masking single tokens. The proportion of masked tokens in each sequence is 15% (by default). Given a sequence with masked tokens $\left\{t_{1},t_{2},\ldots ,t_{M}\right\}$, we calculated the cross-entropy loss as follows:


(1) $$L_{\text{mask}}=\frac{1}{M}\sum_{i=1}^{M}-\text{log}\;\left(\frac{\;\text{exp}({\hat{T}}_{i}[v(t_{i})])}{\sum_{j=1}^{\left|v\right|}\;\text{exp}({\hat{T}}_{i}[j])}\right)$$

where *M* represents the number of masked tokens in the sequence. v(·) denotes the index of a specific token in the token vocabulary, and |v| represents the size of the token vocabulary. ${\hat{T}}_{i}\in R^{\left|v\right|}$ is the output of the token prediction head for *i*th masked token. Given the ATAC-seq dataset (denoted as $D_{0}$), we minimized the cross-entropy loss for masked tokens in the pretraining stage:


(2) Θ0←arg minΘLmask(D0;Θ)

where Θ and $\Theta_{0}$ represent the initial and pretrained model parameters, respectively. Additionally, we randomly sliced sequences in each batch using length ratios drawn uniformly from [0.1, 1.0], enabling TRAFICA to process genomic sequences with varying lengths.

TRAFICA was pretrained over a total of 500 000 steps using a batch size of 128. We employed a warm-up strategy to select the learning rates during the pretraining stage (from 0.0001 to 0.0001 over the first 50 000 steps, cosine decay to 0 in the remaining steps). We applied the AdamW optimizer (Loshchilov and Hutter 2019) with the hyperparameters $\beta_{1}=0.9$, $\beta_{2}=0.98$, and $\epsilon =10^{-7}$ to calculate the gradient and optimize the model parameters. The pretraining process was executed on an Nvidia Tesla A100 GPU card over 5 days.

### 2.4 Model fine-tuning

During the fine-tuning stage, we froze all pretrained parameters and inserted trainable LoRA modules (rank = 12, α=24) exclusively into the query, key, and value projection layers of each multihead self-attention layer. Only the LoRA adapters and the newly added affinity prediction head were updated, enabling TF-specific adaptation while preserving the pretrained open chromatin feature (Fig. 1B). Given a specific TF–DNA binding dataset (denoted as $D^{{\;}^{\prime }}$) with *N* sequences and corresponding affinities $\left\{y_{1},y_{2},\ldots ,y_{N}\right\}$, we minimized the mean squared error (MSE) as follows:


(3) $$\Theta_{\text{LoRA}}^{{\;}^{\prime }},\Theta_{\text{Aff}}^{{\;}^{\prime }}\leftarrow \underset{\Theta_{\text{LoRA}},\Theta_{\text{Aff}}}{\arg\min}L_{\text{MSE}}(D^{{\;}^{\prime }};\Theta_{0},\Theta_{\text{LoRA}},\Theta_{\text{Aff}})$$

(4) $$L_{\text{MSE}}=\frac{1}{N}\sum_{i=1}^{N}{\left({\hat{y}}_{i}-y_{i}\right)}^{2}$$

where $\Theta_{\text{LoRA}}^{{\;}^{\prime }}$ and $\Theta_{\text{Aff}}^{{\;}^{\prime }}$ represent the parameters of the LoRA modules and affinity prediction head fine-tuned on $D^{{\;}^{\prime }}$. $\hat{y_{i}}$ is the output of the affinity prediction head for *i*th sequence.

AdamW optimizer was utilized to optimize the parameters of $\Theta_{\text{LoRA}}$ and $\Theta_{\text{Aff}}$, with a constant learning rate of 0.0001 and a batch size of 128. Early stopping strategy was implemented with a maximum of 300 epochs and a tolerance of 10 epochs by monitoring validation loss. For PBM datasets, we reserved 10% of the fine-tuning data as a validation set through random sampling. For fair benchmarking, all DNA language models (DNABERT, DNABERT2, and Nucleotide Transformer) were optimized using identical fine-tuning paradigms and hyperparameter settings as TRAFICA, except LoRA settings (rank = 8, α=16) to ensure the size of LoRA modules is similar between TRAFICA and the others. The training settings for the comparison tools used are included in Note 7, available as supplementary data at *Bioinformatics* online.

### 2.5 Attention score analysis

We employed TRAFICA (base-level) to compute nucleotide-level attention scores, using the top 1000 sequences ranked by predicted affinity from hold-out test sets for motif derivation. We excluded attention scores from the initial two Transformer-encoder layers, as these early layers capture overly broad sequence features. Subsequently, we averaged layerwise and headwise attention matrices to generate attention score vectors representing the attention score of each nucleotide received from others. High-attention nucleotides were identified as those exceeding the mean attention score threshold. We detected high-attention subsequences containing a minimum of eight consecutive high-attention nucleotides. These subsequences were subjected to sequence alignment, yielding fixed-length subsequences with alignment gaps. The resulting subsequences were used to compute position frequency matrices and derive binding motif logos. There is a slight difference in analyzing head-specific patterns. We averaged layerwise attention matrices to compute head-specific attention score vectors. High-attention regions were identified for each attention head, with subsequent high-attention subsequence detection restricted to these regions, as they represent the most strongly attended positions within each specific head.

## 3 Results

### 3.1 Overview of TRAFICA

#### 3.1.1 Data usage

We initially constructed a comprehensive ATAC-seq dataset, which included 2 846 639 genomic sequences from open chromatin regions in the human genome (Fig. 1A). The open chromatin regions were defined based on the signal peaks in 197 ATAC-seq experiments (Table 1, available as supplementary data at *Bioinformatics* online) from the Encyclopedia of DNA Elements (ENCODE) project (The ENCODE Project Consortium 2012, Luo *et al.* 2020), covering 115 unique cell lines. More details for constructing the ATAC-seq dataset can be found in Note 1, available as supplementary data at *Bioinformatics* online. By intersecting these genomic sequences with annotated regulatory elements from the EnhancerAtlas 2.0 (Gao and Qian 2020) and Eukaryotic Promoter Database (Dreos *et al.* 2013), we found that 93.23% of the pretraining ATAC-seq dataset overlapped with known enhancers and 37.64% with promoters, revealing potential regulatory roles (Note 2, available as supplementary data at *Bioinformatics* online). This established ATAC-seq dataset was then used for TRAFICA pretraining to learn the characteristics of sequences with natural TF binding preferences (Fig. 1A). The length of sequences is up to 512bps in the ATAC-seq dataset.

Moreover, we collected *in vitro* TF-DNA binding profiles measured by PBM and HT-SELEX technologies to fine-tune TRAFICA. PBM profiles were collected from DREAM5 Challenge PBM data source (Weirauch *et al.* 2013), consisting of 132 datasets for 66 TFs (Table 2, available as supplementary data at *Bioinformatics* online). The length of sequences is 36 bps in the PBM datasets. HT-SELEX profiles were generated from three previous HT-SELEX studies: Jolma *et al.* (2013), Yang *et al.* (2017), and Yin *et al.* (2017), which can be accessed in European Nucleotide Archive (ENA). We implemented a quality control to remove low-quality datasets (Note 3.1, available as supplementary data at *Bioinformatics* online), resulting in 440 datasets for 336 TFs (Table 3, available as supplementary data at *Bioinformatics* online). The relative binding affinities of DNA sequences were estimated based on the fold enrichment of subsequences between HT-SELEX selection cycles (Slattery *et al.* 2011) (Fig. 1B and Note 3.2, available as supplementary data at *Bioinformatics* online). The lengths of sequences are 20, 30, and 40 bps in the HT-SELEX datasets. To assess *in vivo* predictive performance, we collected 274 ChIP-seq profiles consisting of 141 TFs across 18 distinct cell lines from the ENCODE project (Note 1 and Table 4, available as supplementary data at *Bioinformatics* online). The length of sequences is up to 100 bps in the ChIP-seq datasets.

#### 3.1.2 Model architecture

The architecture of TRAFICA is built on the vanilla Transformer-encoder structure (Vaswani *et al.* 2017, Devlin *et al.* 2019), which utilizes the self-attention mechanism to capture contextual relationships in sequential data. The model comprises eight Transformer-encoder layers, each consisting of eight attention heads. We implemented RoPE (Su *et al.* 2024) for token position encoding. The number of model parameters is about 26 million. Model configuration is presented in Table 5, available as supplementary data at *Bioinformatics* online. Additionally, a token prediction head (Fig. 1A, available as supplementary data at *Bioinformatics* online) is incorporated to perform masked language modeling in the pretraining stage (Fig. 1A). This token prediction head is discarded after pretraining. Additionally, LoRA modules and an affinity prediction head (Fig. 1B, available as supplementary data at *Bioinformatics* online) are employed to predict TF–DNA binding affinity in the fine-tuning stage (Fig. 1B). More details about the network structure of TRAFICA can be found in Note 4, available as supplementary data at *Bioinformatics* online.

### 3.2 *In vitro* TF–DNA binding affinity prediction on the DREAM5 PBM datasets

**Figure 2.**
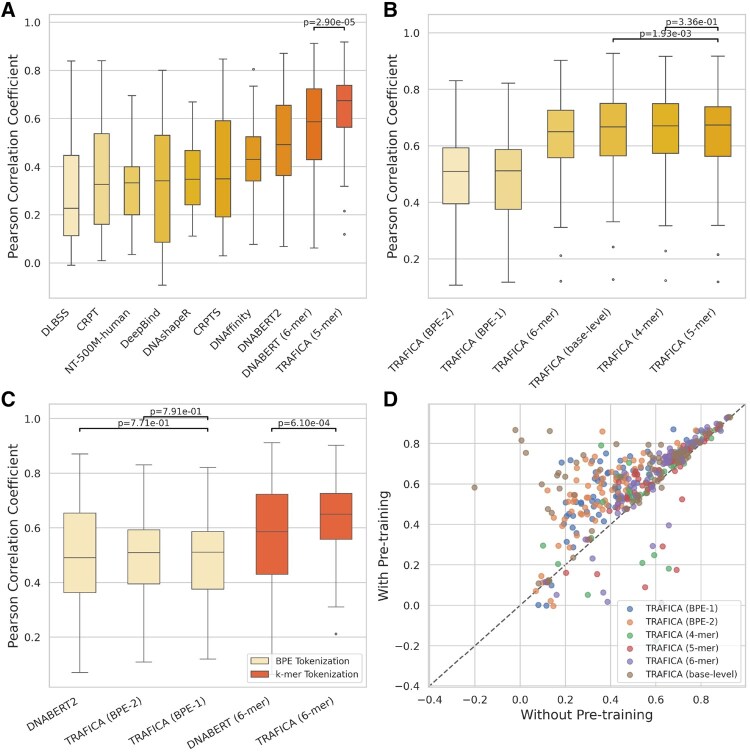
Performance in predicting *in vitro* TF-DNA binding affinity using the DREAM5 PBM datasets. (A) Predictive performance comparison. The *P*-values were calculated by Wilcoxon rank-sum test. (B) Ablation study assessing the impact of diverse DNA sequence tokenization approaches. (C) Effect of sequence types used for model pretraining: sequences from open chromatin regions (TRAFICA) versus from the whole genome (DNABERT/DNABERT2). (D) Ablation study evaluating the pretraining stage of TRAFICA. Each dot represents a TF involved in the PBM datasets. The unit of axis is Pearson correlation coefficient.

The DREAM5 PBM datasets contain two microarrays, which were alternately used as the fine-tuning and test sets for model evaluation (left panel of Fig. 1C): (i) TRAFICA was fine-tuned using the first 33 TFs from the first microarray, and tested on the same TFs from the second microarray and (ii) TRAFICA was fine-tuned using the last 33 TFs from the second microarray and tested on the same TFs from the first microarray. We compared the performance of TRAFICA, DeepBind, DLBSS, CRPT, CRPTS, DNAshapeR, DNAffinity, Nucleotide Transformer (version: NT-500M-human), DNABERT, and DNABERT2. Pearson correlation coefficient (PCC) is utilized as the evaluation metric (Note 5, available as supplementary data at *Bioinformatics* online). TRAFICA (5-mer) achieved the best performance (average PCC = 0.647; Fig. 2A), which significantly outperformed the second-best tool DNABERT (6-mer) (average PCC = 0.549, *P* = 2.9e-05). Additionally, we assessed the effect of tokenization methods in predicting TF–DNA binding affinity by fine-tuning six versions of TRAFICA, each employing a distinct tokenization approach. For BPE tokenization, we employed two BPE vocabularies: (BPE-1) constructing a vocabulary based on the pretraining ATAC-seq dataset and (BPE-2) using the established BPE vocabulary constructed in the study of DNABERT2. The experimental results showed that *k*-mer tokenizations (base-level is equivalent to *k *= 1) consistently outperformed BPE tokenizations (Fig. 2B). Furthermore, we compared the versions of TRAFICA and DNABERT/DNABERT2 with an identical tokenization approach to investigate whether pretraining on open chromatin regions can perform better than on the whole genome. As shown in Fig. 2C, TRAFICA (6-mer) significantly outperformed DNABERT (6-mer) with *P*=6.1e-04, while the performance of TRAFICA (BPE-1), TRAFICA (BPE-2), and DNABERT2 was insignificantly different (average PCCs = 0.490, 0.493, and 0.490). Moreover, we performed an ablation study to investigate if including the pre-training stage could improve performance. Specifically, we compared two configurations: (with pretraining) full fine-tuning of the pretrained TRAFICA without using LoRA modules and (without pretraining) training TRAFICA from scratch. The results indicated a substantial improvement by involving the pretraining stage across all versions of TRAFICA [Fig. 2D; *P* = 6.7e-12 for TRAFICA (BPE-1); 8.37e-12 for TRAFICA (BPE-2); 3.36e-06 for TRAFICA (4-mer); 6.01e-06 for TRAFICA (5-mer); 1.58e-04 for TRAFICA (6-mer); 7.38e-11 for TRAFICA (base-level)]. Notably, TRAFICA (base-level) achieved consistent improvements for almost all TFs, with the average PCC rising from 0.4769 to 0.645. This highlighted the necessity of integrating natural TF binding preferences as prior knowledge in predicting TF–DNA binding affinity.

### 3.3 TF–DNA binding affinity prediction using TRAFICA fine-tuned by HT-SELEX datasets

**Figure 3.**
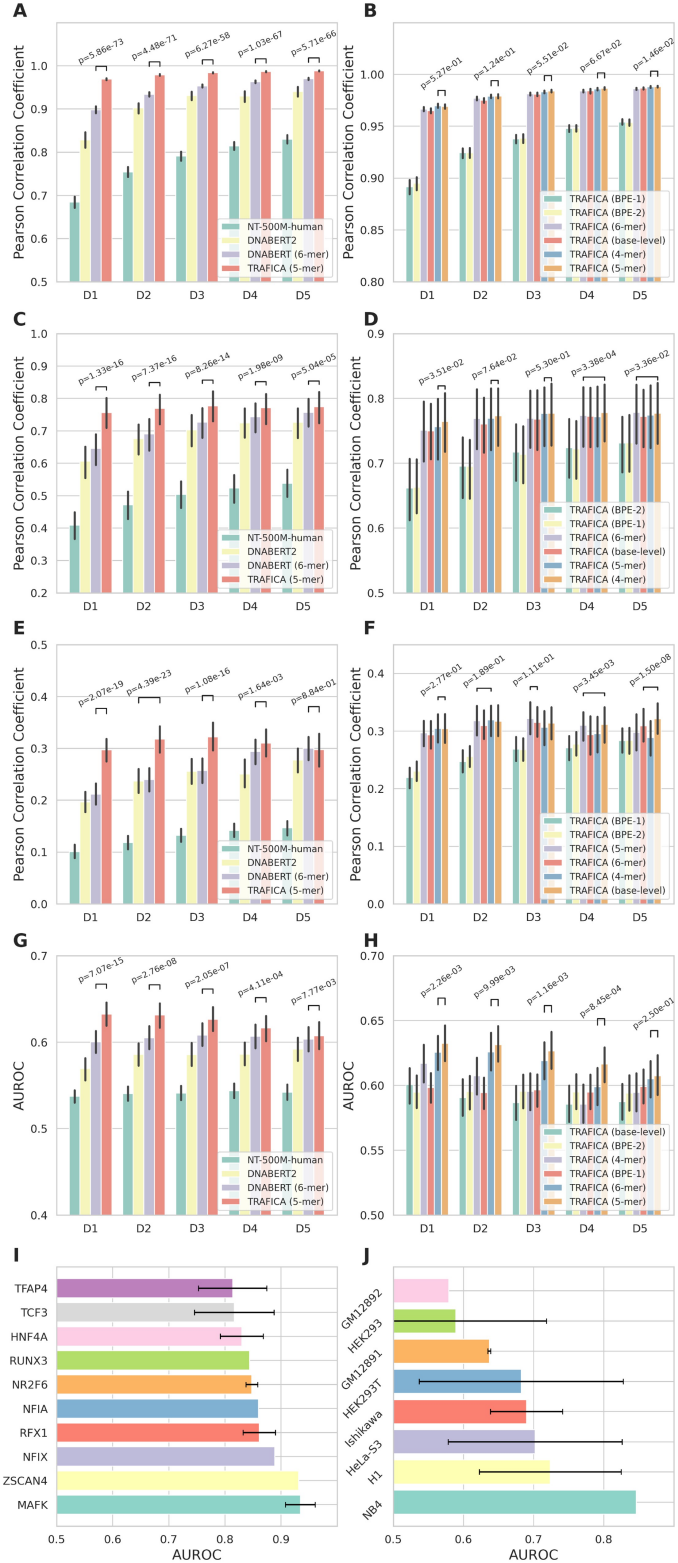
Performance in predicting TF-DNA binding affinity using HT-SELEX fine-tuned models. (A) Performance comparison of hold-out test. *P*-values were calculated by Wilcoxon rank-sum test. Error bars present 95% confidence intervals. (B) Ablation study assessing the impact of diverse DNA sequence tokenization approaches on hold-out test. (C) Performance comparison of cross-experiment test. (D) Impact of DNA tokenization on cross‐experiment test. (E) Performance comparison of cross-platform test. (F) Impact of DNA tokenization on cross‐platform test. (G) Performance comparison of *in vivo* test. (H) Impact of DNA tokenization on *in vivo* test. (I) Top-10 TFs with best predictive performance on ChIP-seq datasets. Error bars present standard deviations. (J) Performance in the cell lines not involved in the pretraining ATAC-seq dataset.

We observed substantial sequence redundancy in HT-SELEX experimental profiles, containing numerous sequences with repetitive patterns (Note 6, available as supplementary data at *Bioinformatics* online). To eliminate redundancy, we sampled sequences rom original HT-SELEX datasets based on their relative binding affinities to create subsets. For each original dataset, we generated five progressively sized subsets (denoted as D1–D5), ranging from 10 000 to 50 000 sequences, to assess the influence of fine-tuning dataset sizes. Additionally, we created hold-out test sets for each subset by sampling sequences from the original datasets (1000 sequences for D1; scaled proportionally up to 5000 for D5). More details can be found in Section 2. The performance in the hold-out test sets showed that TRAFICA (5-mer) was robust and always outperformed those competing tools (Fig. 3A and B), with the average PCCs of 0.969 (D1), 0.979 (D2), 0.984 (D3), 0.986 (D4), and 0.988 (D5). This implies that larger fine-tuning datasets can consistently improve performance in the hold-out test. Next, we evaluated the performance of TRAFICA across different HT-SELEX datasets (cross-experiment test). For the TFs that exist in multiple HT-SELEX datasets, we extracted the fine-tuning set from one dataset and the test set from the other dataset(s). We generated 123 such datasets for 82 TFs in total (Table 6, available as supplementary data at *Bioinformatics* online). TRAFICA (5-mer) consistently outperformed DNABERT, DNABERT2, and Nucleotide Transformer (Fig. 3C), regardless of the number of sequences included in fine-tuning sets. Ablation study for tokenization approaches demonstrated that 4-mer tokenization achieved superior performance compared to others in the cross-experiment test (Fig. 3D). To evaluate the generalizability of TRAFICA across different assay platforms, we fine-tuned the pre-trained models on HT-SELEX datasets and evaluated them on the test datasets from DREAM5 PBM datasets. We identified 166 datasets for 31 TFs (Table 7, available as supplementary data at *Bioinformatics* online), in which the TFs both existed in HT-SELEX and DREAM5 PBM datasets. TRAFICA (5-mer) achieved the best performance compared to other methods across D1–D4 (Fig. 3E), with marginal performance differences relative to DNABERT (6-mer) on D5 (*P* = 0.884). These results indicated that TRAFICA had better generalizability than the existing tools on datasets from different *in vitro* binding assay technologies. We noticed that the average PCC of TRAFICA (5-mer) was higher on D3 compared to D4 and D5, a trend not shown on other models. This performance limitation could be attributed to the model’s reduced capacity (number of model parameters: TRAFICA ≈ 1/4 DNABERT), which becomes insufficient for processing large-scale datasets effectively (e.g. capturing noises from a large amount of sequences instead of binding patterns), ultimately resulting in poor generalizability. Tokenization ablation showed that performance varies across D1–D5 in the cross-platform test (Fig. 3F).

Predicting *in vivo* TF-DNA binding affinity is more challenging than *in vitro* predictions due to the influence of multiple factors (e.g. cobinding mechanism and DNA methylation), which are dynamic in diverse cell status. We fine-tuned TRAFICA on the datasets from *in vitro* HT-SELEX technology and tested the model on datasets from *in vivo* ChIP-seq technology. This strategy allows us to explore whether TRAFICA could leverage the characteristics of sequences from open chromatin regions to improve the prediction of *in vivo* TF binding affinity. This assessment was applied to 389 datasets for 141 TFs (18 cell lines) included in both HT-SELEX and ChIP-seq datasets (Table 8, available as supplementary data at *Bioinformatics* online). The area under the receiver operating characteristic curve (AUROC) was used as the evaluation metric, as the labels of ChIP-seq datasets were binary. TRAFICA (5-mer) significantly outperformed the second-best tool DNABERT (6-mer) in predicting *in vivo* TF-DNA binding affinity across different numbers of sequences used for model fine-tuning (Fig. 3G). We observed that performance declines with the increasing size of fine-tuning datasets, mirroring the similar trend identified in cross-platform testing (Fig. 3E). DNABERT (6-mer) also showed performance decline on large datasets (comparing D3–D4/D5). Tokenization ablation demonstrated that 5-mer tokenization consistently outperformed the others in the *in vivo* test (Fig. 3H). Furthermore, we presented top-10 TFs with best performance on D1 from TRAFICA (5-mer), identifying TFs MAFK (from cell lines: A549, H1, HeLa-S3, HepG2, IMR-90, K562, MCF-7, and GM12878) and ZSCAN4 (HEK293) as the top performers on D1 (mean AUROC>0.9), with TFs RFX1 (HepG2, K562, and MCF-7), NFIX (K562), NR2F6 (HepG2 and K562), RUNX3 (GM12878), HNF4A (HepG2), TCF3 (HepG2, K562, and GM12878), and TFAP4 (HepG2 and K562) also demonstrating strong performance (mean AUROC>0.8) (Fig. 3I). All of the above cell lines, except HEK293, were included in the ATAC-seq pretraining dataset. To assess the generalizability of TRAFICA to cell lines absent from pretraining, we evaluated its capability on eight cell lines from the ChIP-seq datasets. TRAFICA (5-mer) achieved considerable performance (AUROC>0.7) in NB4 (TF: MAX), H1 (TFs: MAFK, RFX5, RXRA, EGR1, SP4, CEBPB, NRF1, MAX, POUF51), and HeLa-S3 (TFs: IR3, MAFK, RFX5, CEPBP, SREBF2, ELK1, NR2C2, NRF1, MAX, and E2F4) cell lines on D1 (Fig. 3J).

### 3.4 Attention-based motif analysis

**Figure 4.**
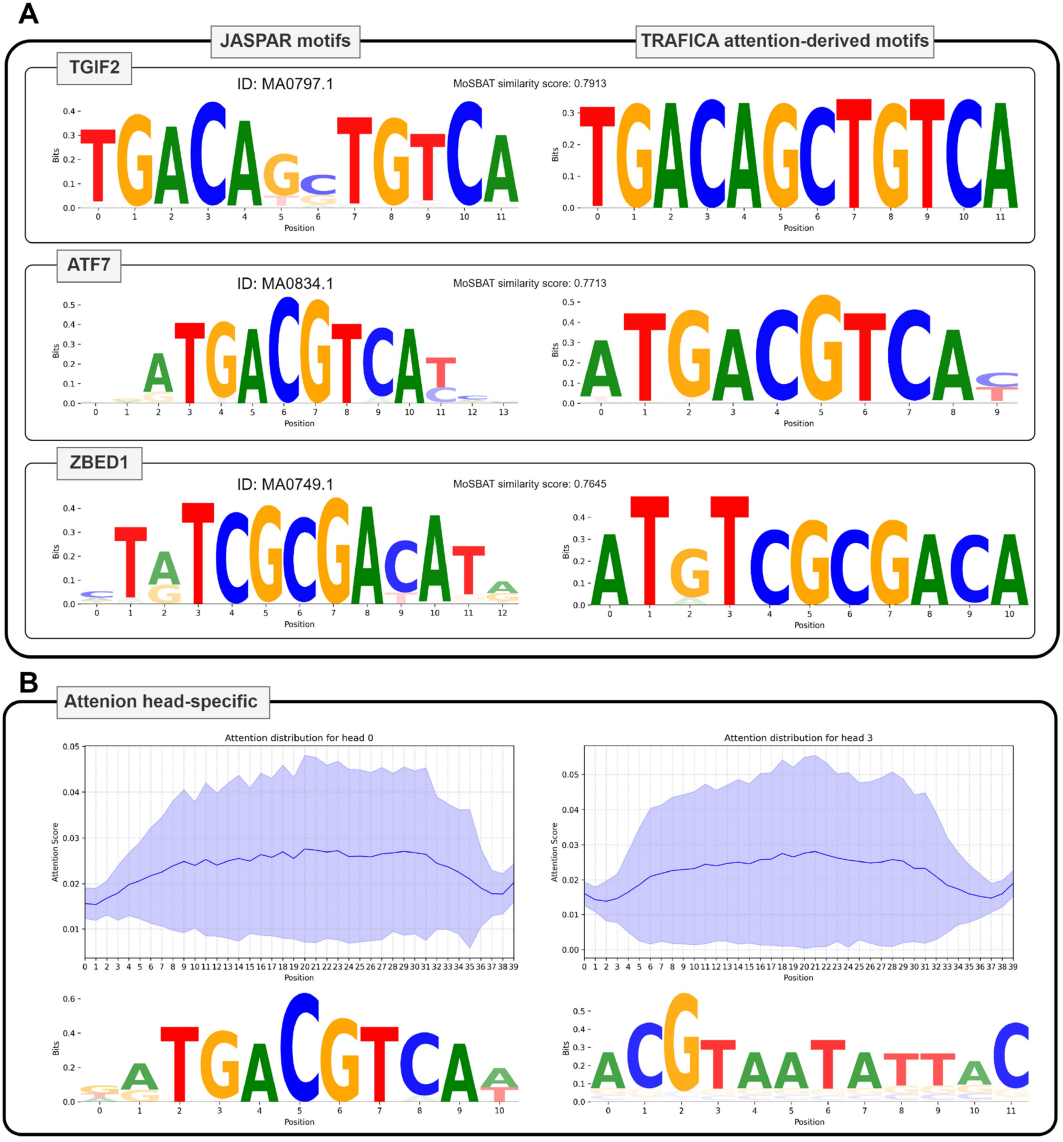
Analysis of binding motifs and attention head-specific patterns. (A) Motif logos of three TFs: TGIF2, ATF7, and ZEBD1. The left panel displays the validated TF binding motifs from the JASPAR database, whereas the right panel presents the TF binding motifs derived from the attention scores. Similarities between each motif pair were calculated using MoSBAT-e scores. (B) Attention head-specific patterns for TF ATF7. The top panel demonstrates the attention score distributions for each position in two different attention heads, whereas the bottom panel shows the corresponding motif logos derived from these heads.

Sequence motifs are typically represented as position frequency matrices or sequence logos that provide an intuitive visualization of TF binding preferences. We employed an attention-based approach (see Section 2) to generate TF binding motifs, interpreting the biological relevance of TRAFICA within its prediction process. We found 242 TFs from 263 TF–motif pairs (Table 9, available as supplementary data at *Bioinformatics* online) with the matching validated motifs in the JASPAR database (Castro-Mondragon *et al.* 2022). We utilized MoSBAT energy (MoSBAT-e) scores (Lambert *et al.* 2016) to quantify the similarity between predicted and validated motifs for the same TFs (motif pairs), and utilized “Logomaker” (Tareen and Kinney 2020) to generate motif logos for visualization. MoSBAT generates random sequences (50 000 by default) and employs two motifs to predict binding scores for these sequences. The correlation between the two sets of predicted scores serves as the motif similarity metric. The logos of the motif pairs with the top-3 highest similarities are shown in Fig. 4A, and their similarities are 0.7913 (TF: TGIF2, JASPAR ID: MA0797.1), 0.7713 (TF: ATF7, JASPAR ID: MA0834.1), and 0.7645 (TF: ZBED1, JASPAR ID: MA0749.1), respectively. The strong agreement among these three pairs explains TRAFICA’s superior performance in predicting TF–DNA binding affinity, suggesting that the model is highly effective in learning biologically relevant TF–DNA binding patterns. We showed motif logos for all 263 motif pairs in Figs 2–5, available as supplementary data at *Bioinformatics* online.

Additionally, we investigated patterns in individual attention heads, revealing heterogeneity among attention heads. Analysis of attention score distributions in randomly selected heads (0 and 3) revealed preferential focus on central positions (e.g. positions 10–30), where significantly higher attention scores were observed (the top panel of Fig. 4B). Although the attention patterns were similar between heads, they processed distinct sequence features (the bottom panel of Fig. 4B). Head 0 captured sequence specificity closely matched the complete ATF7 binding motif, while Head 3 recognized both the ATF7 motif fragment (positions 0–5) and additional sequence characteristics (e.g. GC content).

## 4 Discussion

The accumulation of high-throughput sequencing data on TF–DNA binding (e.g. PBM, HT-SELEX, ChIP-seq, and ATAC-seq) offers a rich landscape for delving into the binding tendencies of TFs and DNA sequences. Although ChIP-seq directly captures genome-wide DNA sequences binding to a specific TF, it has its own limitation (Lambert *et al.* 2018) because antibodies may not always be available for all TFs. Moreover, binding patterns can vary across different cell lines for a TF (Gertz *et al.* 2012), which can skew the understanding of intrinsic TF binding preferences. PBM technology is widely used to measure the TF–DNA binding intensities of synthesized nucleotide sequences to specific TFs by detecting fluorescence signal intensities (Berger and Bulyk 2009) (top-middle panel in Fig. 1B). A majority of sequences designed for PBM experiments may have low binding affinities to the target TFs because the synthetic sequences may not closely match specific TF-binding motifs. HT-SELEX experiments can generate abundant nucleotide sequences with relatively higher binding affinities by multicycle selection (top-left panel in Fig. 1B). Several studies have utilized HT-SELEX technology to investigate TF–DNA binding affinity (Jolma *et al.* 2010, 2013, Yang *et al.* 2017, Yin *et al.* 2017), the interactions of RNA-binding proteins (Jolma *et al.* 2020, Laverty *et al.* 2022), and the interactions of drug-target proteins [e.g. aptamer-binding proteins (Zhou and Rossi 2017)]. Unlike PBM and HT-SELEX, which directly measure TF–DNA binding affinity, ATAC-seq measures chromatin accessibility that provides natural TF binding preferences. For example, promoters and enhancers are usually observed in those highly accessible (open chromatin) genomic regions (Boyle *et al.* 2008) (Note 2, available as supplementary data at *Bioinformatics* online). We pretrained TRAFICA using sequences from open chromatin regions annotated by the peaks from ATAC-seq experiments to enable it to learn natural TF binding preferences. Our results demonstrated that involving the pretraining stage significantly improved the performance of TRAFICA in predicting TF–DNA binding affinity.

Tokenization ablation studies demonstrated that 5-mer tokenization showed superior robustness compared to the others, indicating that TF binding regions may primarily recognize 5-nucleotide core sequences. TRAFICA’s compact architecture (compared to other Transformer-based models: DNABERT/DNABERT2, Nucleotide Transformer) demands fewer computational resources for binding affinity prediction and demonstrates superior performance with limited fine-tuning data. However, the model shows decreased effectiveness when processing large fine-tuning datasets, where noise may obscure true binding patterns.

The scarcity of TF ChIP-seq profiles, particularly for cell-type-specific TF–DNA binding, presents a significant challenge for comprehensive *in vivo* TF–DNA binding studies. Similar to current TF–DNA binding prediction tools and DNA language models, TRAFICA is limited in integrating cell-type-specific sequence characteristics in its analyses. Fortunately, the advancement of single-cell multiomics provides unprecedented opportunities to predict cell-type-specific TF–DNA binding affinity. Several studies (Schep *et al.* 2017, Yuan and Kelley 2022) have utilized single-cell ATAC-seq profiles to explore cell heterogeneity by analyzing cell-type-specific open chromatin regions. In addition, a previous study (Schwessinger *et al.* 2023) utilized promoter sequences of target genes to predict gene expression using single-cell RNA-seq profiles, providing valuable insight into understanding gene regulation. These single-cell multi-omics technologies provide a foundation for developing tools that could potentially predict cell-type-specific TF-DNA binding affinity.

## Supplementary Material

btaf469_Supplementary_Data

## Data Availability

The ATAC-seq profiles and ChIP-seq profiles can be accessed in the ENCODE project (https://www.encodeproject.org/). The DREAM5 PBM experimental data are available at https://hugheslab.ccbr.utoronto.ca/supplementary-data/DREAM5/. The HT-SELEX experimental data can be accessed by the ENA accession ID: PRJEB3289 (Jolma *et al.* 2013), PRJEB14744 (Yang *et al.* 2017), and PRJEB9797/PRJEB20112 (Yin *et al.* 2017). The source code of TRAFICA is available at https://github.com/ericcombiolab/TRAFICA. The pretrained models are available at https://huggingface.co/collections/Allanxu/trafica-6863a46a15f497be8ce4e25a. All fine-tuned LoRAs are available at https://huggingface.co/Allanxu/Finetuned_TRAFICA_All. The processed sequence data of ATAC-seq, HT-SELEX, PBM, and ChIP-seq can be accessed at https://zenodo.org/records/15781226.

## References

[btaf469-B1] Alipanahi B , Delong A, Weirauch MT et al Predicting the sequence specificities of DNA-and RNA-binding proteins by deep learning. Nat Biotechnol 2015;33:831–8. 26213851 10.1038/nbt.3300

[btaf469-B2] Barissi S , Sala A, Wieczór M et al DNAffinity: a machine-learning approach to predict DNA binding affinities of transcription factors. Nucleic Acids Res 2022;50:9105–14. 36018808 10.1093/nar/gkac708 PMC9458447

[btaf469-B3] Berger MF , Bulyk ML. Universal protein-binding microarrays for the comprehensive characterization of the DNA-binding specificities of transcription factors. Nat Protoc 2009;4:393–411. 19265799 10.1038/nprot.2008.195 PMC2908410

[btaf469-B4] Berger MF , Philippakis AA, Qureshi AM et al Compact, universal DNA microarrays to comprehensively determine transcription-factor binding site specificities. Nat Biotechnol 2006;24:1429–35. 16998473 10.1038/nbt1246 PMC4419707

[btaf469-B5] Boyle AP , Davis S, Shulha HP et al High-resolution mapping and characterization of open chromatin across the genome. Cell 2008;132:311–22. 18243105 10.1016/j.cell.2007.12.014 PMC2669738

[btaf469-B6] Buenrostro JD , Wu B, Chang HY et al ATAC-seq: a method for assaying chromatin accessibility genome-wide. Curr Protoc Mol Biol 2015;109:21–9. 10.1002/0471142727.mb2129s109 PMC4374986 25559105

[btaf469-B7] Castro-Mondragon JA , Riudavets-Puig R, Rauluseviciute I et al Jaspar 2022: the 9th release of the open-access database of transcription factor binding profiles. Nucleic Acids Res 2022;50:D165–73. 34850907 10.1093/nar/gkab1113 PMC8728201

[btaf469-B8] The ENCODE Project Consortium. An integrated encyclopedia of DNA elements in the human genome. Nature 2012;489:57. 22955616 10.1038/nature11247 PMC3439153

[btaf469-B9] Dalla-Torre H , Gonzalez L, Mendoza-Revilla J et al Nucleotide transformer: building and evaluating robust foundation models for human genomics. Nat Methods 2025;22:287–97. 39609566 10.1038/s41592-024-02523-z PMC11810778

[btaf469-B10] Devlin J , Chang M-W, Lee K et al Bert: pre-training of deep bidirectional transformers for language understanding. In: *Proceedings of the 2019 conference of the North American chapter of the association for computational linguistics: human language technologies*, volume 1 (long and short papers),**2019*.*

[btaf469-B11] Dreos R , Ambrosini G, Cavin Périer R et al EPD and EPDnew, high-quality promoter resources in the next-generation sequencing era. Nucleic Acids Res 2013;41:D157–64. 23193273 10.1093/nar/gks1233 PMC3531148

[btaf469-B12] Gao T , Qian J. Enhanceratlas 2.0: an updated resource with enhancer annotation in 586 tissue/cell types across nine species. Nucleic Acids Res 2020;48:D58–64. 31740966 10.1093/nar/gkz980 PMC7145677

[btaf469-B13] Gertz J , Reddy TE, Varley KE et al Genistein and bisphenol a exposure cause estrogen receptor 1 to bind thousands of sites in a cell type-specific manner. Genome Res 2012;22:2153–62. 23019147 10.1101/gr.135681.111 PMC3483545

[btaf469-B14] Hu EJ , Shen Y, Wallis P et al Lora: low-rank adaptation of large language models. In: *The International Conference on Learning Representations*, Vol. 1, 2022, 3.

[btaf469-B15] Ji Y , Zhou Z, Liu H et al DNABERT: pre-trained bidirectional encoder representations from transformers model for DNA-language in genome. Bioinformatics 2021;37:2112–20. 33538820 10.1093/bioinformatics/btab083 PMC11025658

[btaf469-B16] Johnson AD. Molecular mechanisms of cell-type determination in budding yeast. Curr Opin Genet Dev 1995;5:552–8. 8664541 10.1016/0959-437x(95)80022-0

[btaf469-B17] Johnson DS , Mortazavi A, Myers RM et al Genome-wide mapping of in vivo protein-DNA interactions. Science 2007;316:1497–502. 17540862 10.1126/science.1141319

[btaf469-B18] Jolma A , Kivioja T, Toivonen J et al Multiplexed massively parallel SELEX for characterization of human transcription factor binding specificities. Genome Res 2010;20:861–73. 20378718 10.1101/gr.100552.109 PMC2877582

[btaf469-B19] Jolma A , Yan J, Whitington T et al DNA-binding specificities of human transcription factors. Cell 2013;152:327–39. 23332764 10.1016/j.cell.2012.12.009

[btaf469-B20] Jolma A , Zhang J, Mondragón E et al Binding specificities of human RNA-binding proteins toward structured and linear RNA sequences. Genome Res 2020;30:962–73. 32703884 10.1101/gr.258848.119 PMC7397871

[btaf469-B21] Kudo T , Richardson J. Sentencepiece: a simple and language independent subword tokenizer and detokenizer for neural text processing. arXiv, arXiv:1808.06226, 2018, preprint: not peer reviewed.

[btaf469-B22] Lambert SA , Albu M, Hughes TR et al Motif comparison based on similarity of binding affinity profiles. Bioinformatics 2016;32:3504–6. 27466627 10.1093/bioinformatics/btw489 PMC5181567

[btaf469-B23] Lambert SA , Jolma A, Campitelli LF et al The human transcription factors. Cell 2018;172:650–65. 29425488 10.1016/j.cell.2018.01.029 PMC12908702

[btaf469-B24] Laverty KU , Jolma A, Pour SE et al Priesstess: interpretable, high-performing models of the sequence and structure preferences of RNA-binding proteins. Nucleic Acids Res 2022;50:e111. 36018788 10.1093/nar/gkac694 PMC9638913

[btaf469-B25] Leinonen R , Akhtar R, Birney E et al The European nucleotide archive. Nucleic Acids Res 2011;39:D28–31. 20972220 10.1093/nar/gkq967 PMC3013801

[btaf469-B26] Li J , Sagendorf JM, Chiu T-P et al Expanding the repertoire of DNA shape features for genome-scale studies of transcription factor binding. Nucleic Acids Res 2017;45:12877–87. 29165643 10.1093/nar/gkx1145 PMC5728407

[btaf469-B27] Loshchilov I , Hutter F. Decoupled weight decay regularization. In: *International Conference on Learning Representations*, 2019.

[btaf469-B28] Loupe JM , Anderson AG, Rizzardi LF et al Multiomic profiling of transcription factor binding and function in human brain. Nat Neurosci 2024;27:1387–99. 38831039 10.1038/s41593-024-01658-8 PMC12991031

[btaf469-B29] Luo Y , Hitz BC, Gabdank I et al New developments on the encyclopedia of DNA elements (encode) data portal. Nucleic Acids Res 2020;48:D882–9. 31713622 10.1093/nar/gkz1062 PMC7061942

[btaf469-B30] Oli V , Gupta R, Kumar P. Foxo and related transcription factors binding elements in the regulation of neurodegenerative disorders. J Chem Neuroanat 2021;116:102012. 34400291 10.1016/j.jchemneu.2021.102012

[btaf469-B31] Orenstein Y , Shamir R. A comparative analysis of transcription factor binding models learned from PBM, HT-SELEX and CHIP data. Nucleic Acids Res 2014;42:e63. 24500199 10.1093/nar/gku117 PMC4005680

[btaf469-B32] Schep AN , Wu B, Buenrostro JD et al Chromvar: inferring transcription-factor-associated accessibility from single-cell epigenomic data. Nat Methods 2017;14:975–8. 28825706 10.1038/nmeth.4401 PMC5623146

[btaf469-B33] Schwessinger R , Deasy J, Woodruff RT et al Single-cell gene expression prediction from DNA sequence at large contexts. bioRxiv, 2023, preprint: not peer reviewed.

[btaf469-B34] Sennrich R , Haddow B, Birch A. Neural machine translation of rare words with subword units. In: Erk K, Smith NA (eds), Proceedings of the 54th Annual Meeting of the Association for Computational Linguistics (Volume 1: Long Papers), p.1715–25. Berlin, Germany: Association for Computational Linguistics, 2016.

[btaf469-B35] Slattery M , Riley T, Liu P et al Cofactor binding evokes latent differences in DNA binding specificity between hox proteins. Cell 2011;147:1270–82. 22153072 10.1016/j.cell.2011.10.053 PMC3319069

[btaf469-B36] Su J , Ahmed M, Lu Y et al Roformer: enhanced transformer with rotary position embedding. Neurocomputing (Amst) 2024;568:127063.

[btaf469-B37] Tareen A , Kinney JB. Logomaker: beautiful sequence logos in python. Bioinformatics 2020;36:2272–4. 31821414 10.1093/bioinformatics/btz921 PMC7141850

[btaf469-B38] Vaswani A , Shazeer N, Parmar N et al Attention is all you need. Adv Neural Inf Process Syst 2017;30.

[btaf469-B39] Wang S , Zhang Q, Shen Z et al Predicting transcription factor binding sites using DNA shape features based on shared hybrid deep learning architecture. Mol Ther Nucleic Acids 2021;24:154–63. 33767912 10.1016/j.omtn.2021.02.014 PMC7972936

[btaf469-B40] Weirauch MT , Cote A, Norel R et al; DREAM5 Consortium. Evaluation of methods for modeling transcription factor sequence specificity. Nat Biotechnol 2013;31:126–34. 23354101 10.1038/nbt.2486 PMC3687085

[btaf469-B41] Yang L , Orenstein Y, Jolma A et al Transcription factor family-specific DNA shape readout revealed by quantitative specificity models. Mol Syst Biol 2017;13:910. 28167566 10.15252/msb.20167238 PMC5327724

[btaf469-B42] Yin Y , Morgunova E, Jolma A et al Impact of cytosine methylation on DNA binding specificities of human transcription factors. Science 2017;356:eaaj2239. 28473536 10.1126/science.aaj2239 PMC8009048

[btaf469-B43] Yuan H , Kelley DR. Scbasset: sequence-based modeling of single-cell ATAC-seq using convolutional neural networks. Nat Methods 2022;19:1088–96. 35941239 10.1038/s41592-022-01562-8

[btaf469-B44] Zhang Q , Shen Z, Huang D-S. Predicting in-vitro transcription factor binding sites using DNA sequence+ shape. IEEE/ACM Trans Comput Biol Bioinform 2021;18:667–76. 31634140 10.1109/TCBB.2019.2947461

[btaf469-B45] Zhou J , Rossi J. Aptamers as targeted therapeutics: current potential and challenges. Nat Rev Drug Discov 2017;16:181–202. 27807347 10.1038/nrd.2016.199 PMC5700751

[btaf469-B46] Zhou Z , Ji Y, Li W et al Dnabert-2: efficient foundation model and benchmark for multi-species genome. In: *International Conference on Learning Representations*, 2024.

