# Supplementary material for: TRAFICA: an open chromatin language model to improve transcription factor binding affinity prediction
Source: Bioinformatics. 2025 Aug 23;41(11):btaf469. doi: 10.1093/bioinformatics/btaf469 (PMC12582366; doi:10.1093/bioinformatics/btaf469)
Supplement: btaf469_Supplementary_Data [file btaf469_supplementary_data.zip › TRAFICA_Bioinformatics_Supplementary_RevisionFinal.pdf]

Supplementary File for *TRAFICA: An Open Chromatin Language Model to Improve Transcription Factor Binding Affinity Prediction*

Yu Xu[1], Chonghao Wang[1], Ke Xu[1], Yi Ding[1], Aiping Lyu[2,*], and Lu Zhang[1,3,*]

[1]Department of Computer Science, Hong Kong Baptist University
[2]School of Chinese Medicine, Hong Kong Baptist University
[3]Institute for Research and Continuing Education, Hong Kong Baptist University
[*]Corresponding author: aipinglu@hkbu.edu.hk, ericluzhang@hkbu.edu.hk

Supplementary Notes

Supplementary Note 1: Data processing for ATAC-seq and ChIP-seq profiles

ATAC-seq We obtained 197 ATAC-seq narrowPeak files for 115 cell lines from the ENCODE project (`https://www.encodeproject.org/`), retaining only peaks with 'pValue' (`https://genome.ucsc.edu/FAQ/FAQformat.html#format12`) larger than 100. For cell lines with multiple replicates, we merged overlapping peaks while preserving the peak with the most significant p-value in each overlapping region, resulting in 2,846,639 ATAC-seq peaks. For each retained peak, we extracted genomic sequences spanning 255 base pairs (bps) upstream to 256 bps downstream of peak summits. We truncated the sequences for peaks exceeding this 512-bps window.

ChIP-seq We collected ChIP-seq narrowPeak files for cell lines from the ENCODE project, initially filtering out profiles containing fewer than 1,000 peaks. For a TF with multiple replicates in an identical cell line, we retained the replicate with the largest number of peaks. For each selected profile, we selected the top 10,000 peaks ranked by 'signalValue' (`https://genome.ucsc.edu/FAQ/FAQformat.html#format12`) as positive sequences, subsequently generating negative sequences through dinucleotide shuffling (Alipanahi et al. 2015) of each positive sequence.

Supplementary Note 2: Potential regulatory roles of open chromatin regions

We obtained 29,598 human promoters from EPDnew (`https://epd.expasy.org/epd/`) and 193,218 enhancers from EnhancerAtlas 2.0 (`http://www.enhanceratlas.org/`). Genomic coordinate analysis revealed that 1,071,667 (37.64%) of the 2,846,639 ATAC-seq peaks overlapped with promoters, while 2,654,026 (93.23%) overlapped with enhancer regions.

Supplementary Note 3: Processing of HT-SELEX profiles

We downloaded the following HT-SELEX experimental data files from the ENA (Leinonen et al. 2010): 1. Jolma et al. (Jolma et al. 2013): 547 HT-SELEX experiments covering 461 unique human and mouse TFs; 2. Yang et al. (Yang et al. 2017): 521 HT-SELEX experiments covering 445 unique human and mouse TFs; and 3. Yin et al. (Yin et al. 2017): 2265 HT-SELEX experiments covering 550 unique human TFs.

3.1: Quality control for HT-SELEX experiments

We filtered out the experiments that lacked the following information: Ensembl ID, structural domains, and amino acid sequences. To ensure the reliability of HT-SELEX experiments, we filtered out low-quality experiments by conducting quality control. Given an HT-SELEX experiment with N selection cycles, we implemented quality control as follows:

1. For i-th ($i \neq 0$) selection cycle, we counted the frequency of all possible 8-mers, denoting as $F_i = \{f_i^1, ..., f_i^{65536}\}$ ($65536 = 4^8$: the number of all 8-mers).

2. For the initial cycle, we estimated the expected frequency of all possible 8-mers by using a fifth-order Markov model, denoting as $E_0 = \{f_0^1, ..., f_0^{65536}\}$. The Markov model was implemented using the R programming package "SELEX" (Rastogi et al. 2022).

3. Following the previous study (Slattery et al. 2011), we defined the ratio of F_i to E_0 as the enrichment score of 8-mers in the i-th cycle, denoting as $ES_i = \{\frac{f_i^1}{f_0^1}, ..., \frac{f_i^{65536}}{f_0^{65536}}\}$).

4. Following the previous studies (Yuan et al. 2019; Barissi et al. 2022), we excluded the experiment if it satisfies one of the following criteria: (a) The Spearman's rank correlation coefficient of the enrichment scores ES_3 and ES_4 is less than 0.8, which implies the neighboring cycles are uncorrelated in the enrichment patterns; (b) The number of sequences with counts larger than 10 in the N-th (final) selection cycle is less than 500; (c) The count of each possible 8-mer in the initial cycle is less than 100, which could result in inaccurate estimates of E_0.

After implementing the above steps, 440 high-quality experiments remained, consisting of 336 unique TFs.

3.2: Relative binding affinity estimation for HT-SELEX data

For each high-quality HT-SELEX experiment, we utilized the R programming package "SELEX" (Rastogi et al. 2022) to estimate relative k-mer binding affinities. We employed a searching range of $\{3, 4, ..., 13, 14\}$ for the optimal hyper-parameter k, as the minimum sequence length of the HT-SELEX datasets used in this study is 14. We then merged the estimated k-mer binding affinities for each sequence to obtain sequence affinities. Given a sequence a, we merge the k-mer binding affinities $A_{\text{k-mer}}$ into the sequence affinity $A_{\text{seq}}(a)$ by the following:

$$A_{\text{seq}}(a) = \frac{\sum_{i=1}^{K} A_{\text{k-mer}_i}}{\sqrt{K}} \tag{1}$$

where K represents the number of k-mers in the sequence a. This approach (Eq. 1) was introduced in the previous study (Jiang et al. 2014) that integrated the statistics of k-mers to generate relative sequence binding affinities. Additionally, we removed the low-complexity sequences that present a DUST (Morgulis et al. 2006) score larger than 2, as low-complexity sequences may consist of repeated k-mers with a high relative k-mer binding affinity. This could cause inaccurate sequence binding affinity. Given a sequence a with a length $n > 2$, its DUST score $S(a)$ is formulated as follows:

$$S(a) = \frac{\sum_{t \in R} c_t(a)(c_t(a) - 1)/2}{l - 1} \tag{2}$$

where R is a set of 64 possible triplets of DNA base combinations (e.g., AAA, AAT, ATT,...). $l = n - 2$ is the number of triplets in the sequence a. $c_t(a)$ represents the frequency of occurrence of the triplet t within the sequence a. Sequences and corresponding binding affinities from the final selection cycles are used for model fine-tuning, as the final cycle typically contains a higher ratio of high-affinity sequences than the early cycles.

Supplementary Note 4: Model structure of TRAFICA

4.1: Embedding Module

Embedding module consists of a token embedding layer and a positional embedding layer. The token embedding layer is a fully connected network, denoted as $W_t \in \mathbb{R}^{d_{\text{vocab}} \times d}$, where d_{vocab} represents the size of the token vocabulary. An input sequence is tokenized as a list of tokens and two specific tokens '[CLS]' and '[SEP]' at both ends (denoted total length as l). Each token t and the corresponding position index p are converted as two one-hot encoding vectors $x_t \in \mathbb{R}^{d_{\text{vocab}}}$ and $x_p \in \mathbb{R}^{l_{\text{max}}}$ (l_{max}: maximum number of input tokens), respectively. The process of the embedding layers can be formulated as follows:

$$h_t = x_t^T W_t \oplus x_p^T W_p \tag{3}$$

$$H^0 = \text{Concat}(h_{\text{cls}}, h_1, h_2, ...h_{l-2}, h_{\text{sep}}) \tag{4}$$

where $h_i \in \mathbb{R}^d$ is the hidden embedding capturing the semantic and positional information of the token t_i, and d denotes the dimension of the hidden embedding. W_p is positional embedding layer, implemented using Rotary Positional Embedding ((Su et al. 2024)). Concat$(\cdot)$ is the operation of concatenation. The matrix $H^0 \in \mathbb{R}^{l \times d}$ of the sequence is the input for the first Transformer-encoder layer.

4.2: Transformer-encoder

The structure of the Transformer-encoder is composed of four components: a multi-head self-attention module, a feed-forward module, layer normalization, and residual connections. Each Transformer-encoder layer takes as input the output from the previous one, except the first layer. Given the Transformer-encoder block with n_{head} attention heads, the dimension of hidden representation $d_k = d/n_{\text{head}}$ in each head, and an input matrix $X \in \mathbb{R}^{l \times d}$, the process of the multi-head self-attention module can be described as follows:

$$\text{MultiHead}(X) = \text{Concat}(\text{AttHead}_1, \text{AttHead}_2, ...\text{AttHead}_{n_{\text{head}}})W^O \tag{5}$$

where $W^O \in \mathbb{R}^{d \times d}$ is the learnable parameters used to aggregate the outputs of attention heads. The self-attention mechanism for each head is defined as follows:

$$\text{AttHead}_i = \text{Softmax}(\frac{XW_i^Q \otimes (XW_i^K)^T}{\sqrt{d_k}})XW_i^V \tag{6}$$

where $\{W_i^Q \in \mathbb{R}^{d \times d_k}, W_i^K \in \mathbb{R}^{d \times d_k}, W_i^V \in \mathbb{R}^{d \times d_k}\}_{i=1}^{n_{\text{head}}}$ are the learnable parameters of the linear projection for each attention head, and $\sqrt{d_k}$ is the scaling factor. $\otimes$ denotes the operation of dot-production. Given an input $X \in \mathbb{R}^{l \times d}$, the feed-forward module is formulated as follows:

$$FNN(X) = \text{GELU}(XW_1^{FNN} + b_1^{FNN})W_2^{FNN} + b_2^{FNN} \tag{7}$$

where $\{W_1^{FNN} \in \mathbb{R}^{d \times d_{FNN}}, b_1^{FNN} \in \mathbb{R}^{d_{FNN}}, W_2^{FNN} \in \mathbb{R}^{d_{FNN} \times d}, b_2^{FNN} \in \mathbb{R}^{d}\}$ are the learnable parameters of the first and second fully connected layers in this module. d_{FNN} represents the dimension of immediate embeddings in the feed-forward module. $\text{GELU}(\cdot)$ is the non-linear activation function. Based on **Equation 5** and **7**, the forward propagation of the Transformer-encoder layers is depicted as follows:

$$H_1 = \text{LayerNorm}(\text{MultiHead}(H) \oplus H) \tag{8}$$

$$H_2 = \text{LayerNorm}(FNN(H_1) \oplus H_1) \tag{9}$$

where $H \in \mathbb{R}^{l \times d}$ represents the output from the previous layer ($H = H^0$ for the first layer and $H = H_2^{i-1}$ for the i-th layer). $\oplus$ denotes the operation of the element-wise addition.

4.3: Token prediction head

Token prediction head consists of two fully connected layers with a GELU activation function and layer normalization for performing masked token prediction in the pre-training phase. Given the output of the last transformer-encoder block H^N, the operation of the token predictor is defined as follows:

$$\hat{T} = \text{LayerNorm}(\text{GELU}(H^{n_{\text{layers}}} W_1^t + b_1^t)) W_2^t + b_2^t \tag{10}$$

where $\{W_1^t \in \mathbb{R}^{d \times d}, b_1^t \in \mathbb{R}^{d}, W_2^t \in \mathbb{R}^{d \times |v|}, b_2^t \in \mathbb{R}^{|v|}\}$ are the parameters of the predictor. $H^{n_{\text{layers}}} \in \mathbb{R}^{l \times d}$ represents the output of the last transformer block. The predicted scores are denoted as $\hat{T} \in \mathbb{R}^{l \times |v|}$, where $|v|$ is the size of the token vocabulary.

4.4: Affinity prediction head

We employed two fully connected layers as a regression predictor to transform the sequence embedding h_{cls} into a scalar value of the predicted affinity $\hat{y}$. The process of this head is shown as follows:

$$\hat{y} = \text{Dropout}(\text{GELU}((\text{Dropout}(h_{\text{cls}}^{n_{\text{layers}}}) W_1^{\text{Aff}} + b_1^{\text{Aff}}))) W_2^{\text{Aff}} + b_2^{\text{Aff}} \tag{11}$$

where $\{W_1^{\text{Aff}} \in \mathbb{R}^{d \times \lfloor d/2 \rfloor}, b_1^{\text{Aff}} \in \mathbb{R}^{\lfloor d/2 \rfloor}, W_2^{\text{Aff}} \in \mathbb{R}^{\lfloor d/2 \rfloor \times 1}, b_2^{\text{Aff}} \in \mathbb{R}^{1}\}$ are the parameters of the prediction head. The probability of Dropout is 0.1.

Supplementary Note 5: Evaluation metrics

Pearson correlation coefficient (PCC) is utilized to evaluate the performance in predicting *in vitro* TF-DNA binding affinity. Given a set of input sequences $X = \{x_1, x_2, ..., x_n\}$ with the label $Y = \{y_1, y_2, ..., y_n\}$ and the predicted affinities $f(X) = \{f(x_1), f(x_2), ..., f(x_n)\}$, the computation of these two metrics is formulated as follows:

$$PCC(Y, f(X)) = \frac{\sum_{i=1}^{n}((y_i - \bar{y})(f(x_i) - \overline{f(x)}))}{\sqrt{\sum_{i=1}^{n}(y_i - \bar{y})^2 \sum_{i=1}^{n}(f(x_i) - \overline{f(x)})^2}} \quad (12)$$

where f and n represent the prediction model and the number of sequences in this set, respectively. $\overline{f(x)} = \frac{1}{n}\sum_{i=1}^{n} f(x_i)$ is the mean value of predicted affinities.

Additionally, AUROC is utilized to evaluate the performance of predicting *in vivo* TF-DNA binding affinity on ChIP-seq datasets, considering the binary nature of labels in ChIP-seq datasets.

Supplementary Note 6: Sequences with repetitive patterns in HT-SELEX datasets

To quantify redundancy, we applied a pipeline combining k-mer counting, Principle Component Analysis (PCA; `https://scikit-learn.org/stable/modules/generated/sklearn.decomposition.PCA.html/`), and Hierarchical Density-Based Spatial Clustering of Applications with Noise (HDBSCAN; `https://scikit-learn.org/stable/modules/generated/sklearn.cluster.HDBSCAN.html/`).

1. For each sequence in an HT-SELEX dataset, we counted their 5-mer frequency vectors ($\mathbb{R}^{1024}$)
2. PCA was employed to reduce the dimension of 5-mer vectors to 50
3. We then applied HDBSCAN (min_cluster_size=50; metric='euclidean') to cluster sequences based PCA results.
4. We calculated the ratio between the number of sequences in the largest cluster and the total number of sequences to quantify sequence redundancy.

The analysis revealed that 365 (83.0%) of 440 HT-SELEX datasets showed the largest cluster ratio greater than 0.7.

Supplementary Note 7: Training settings for comparison tools

We followed the default training setting of CRPTS (Wang et al. 2021) to train deep learning-based tools (DeepBind, DLBSS, CRPT, and CRPTS), using an Adadelta optimizer with a learning rate of 0.01, a batch size of 300, and a maximum of 100 epochs. The parameters of the Adadelta optimizer and the dropout rate in neural network layers were sampled within the pre-defined range (Delta:{0.9, 0.99, 0.999}; Moment: {1e-04, 1e-06, 1e-08}; Dropout:{0.2,0.5}). The training procedure for each dataset was repeated 20 times to select the optimal parameters of the Adadelta optimizer and the dropout rate. For training on HT-SELEX subsets, we adjusted the learning rate to 0.1, as we observed that the models could not be optimal during 100 epochs using the default setting. We utilized the R programming package "DNAshapeR" to extract the DNA shape features from sequences, and used the Python programming package "scikit-learn" (Pedregosa et al. 2011) ("sklearn.ensemble.RandomForestRegressor" with the default parameters) to implement the random forest model for TF-DNA binding affinity prediction. Regarding the implementation of DNAffinity, we applied its default setting to train and test models.

References of Supplementary Notes

Alipanahi, Babak et al. (2015). “Predicting the sequence specificities of DNA-and RNA-binding proteins by deep learning”. In: *Nature biotechnology* 33.8, pp. 831–838.

Barissi, Sandro et al. (2022). “DNAffinity: a machine-learning approach to predict DNA binding affinities of transcription factors”. In: *Nucleic Acids Research* 50.16, pp. 9105–9114.

Jiang, Peng et al. (2014). “MPBind: a Meta-motif-based statistical framework and pipeline to Predict Binding potential of SELEX-derived aptamers”. In: *Bioinformatics* 30.18, pp. 2665–2667.

Jolma, Arttu et al. (2013). “DNA-binding specificities of human transcription factors”. In: *Cell* 152.1-2, pp. 327–339.

Leinonen, Rasko et al. (2010). “The European nucleotide archive”. In: *Nucleic acids research* 39.suppl_1, pp. D28–D31.

Morgulis, Aleksandr et al. (2006). “A fast and symmetric DUST implementation to mask low-complexity DNA sequences”. In: *Journal of Computational Biology* 13.5, pp. 1028–1040.

Pedregosa, Fabian et al. (2011). “Scikit-learn: Machine learning in Python”. In: *the Journal of machine Learning research* 12, pp. 2825–2830.

Rastogi, C et al. (2022). “SELEX: Functions for analyzing SELEX-seq data. R package version 1.30.0”. In: *Bioconductor*.

Slattery, Matthew et al. (2011). “Cofactor binding evokes latent differences in DNA binding specificity between Hox proteins”. In: *Cell* 147.6, pp. 1270–1282.

Su, Jianlin et al. (2024). “Roformer: Enhanced transformer with rotary position embedding”. In: *Neurocomputing* 568, p. 127063.

Wang, Siguo et al. (2021). “Predicting transcription factor binding sites using DNA shape features based on shared hybrid deep learning architecture”. In: *Molecular Therapy-Nucleic Acids* 24, pp. 154–163.

Yang, Lin et al. (2017). “Transcription factor family-specific DNA shape readout revealed by quantitative specificity models”. In: *Molecular systems biology* 13.2, p. 910.

Yin, Yimeng et al. (2017). “Impact of cytosine methylation on DNA binding specificities of human transcription factors”. In: *Science* 356.6337, eaaj2239.

Yuan, Han et al. (2019). “BindSpace decodes transcription factor binding signals by large-scale sequence embedding”. In: *Nature methods* 16.9, pp. 858–861.

Supplementary Tables

- Supplementary Table S1: Details of the collected ENCODE ATAC-seq experiments
- Supplementary Table S2: Details of the collected PBM experiments
- Supplementary Table S3: Details of the collected HT-SELEX experiments
- Supplementary Table S4: Details of the collected ChIP-seq experiments
- Supplementary Table S5: Model configuration of TRAFICA
- Supplementary Table S6: Datasets for cross-experiment test
- Supplementary Table S7: Datasets for cross-platform test
- Supplementary Table S8: Datasets for *in vivo* test
- Supplementary Table S9: Motif comparisons

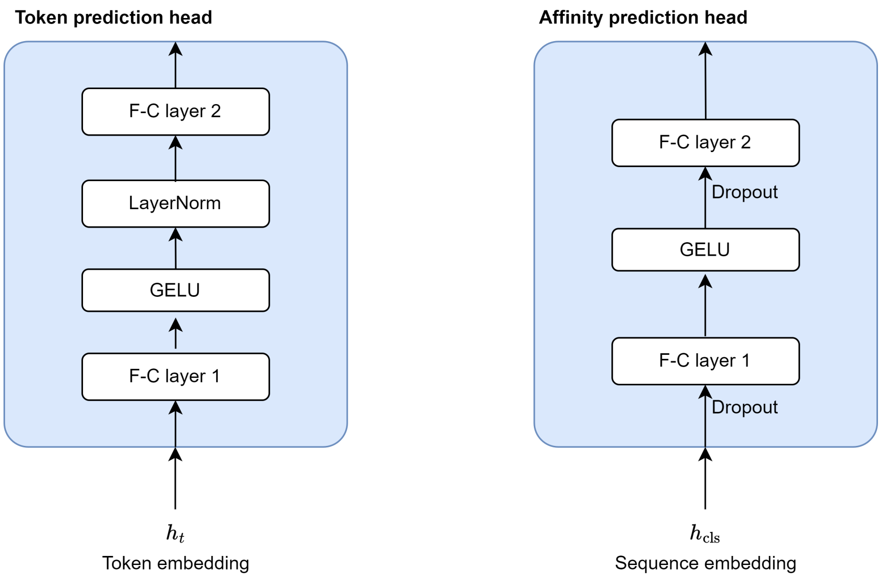

Figure S1. The architectures of token and affinity prediction heads. **(A)** Token prediction head consists of 4 sub-components, including two fully connected (F-C) layers ($\{W_1^t \in \mathbb{R}^{d\times d}, b_1^t \in \mathbb{R}^d\}$, $\{W_2^t \in \mathbb{R}^{d\times|v|}, b_2^t \in \mathbb{R}^{|v|}\}$), a GELU activation, and LayerNorm. h_t represents each token embedding output from the final Transformer-encoder layer. **(B)** Affinity prediction head comprises two F-C layers ($\{W_1^{\text{Aff}} \in \mathbb{R}^{d\times d}, b_1^{\text{Aff}} \in \mathbb{R}^d, W_2^{\text{Aff}} \in \mathbb{R}^{d\times 1}, b_2^{\text{Aff}} \in \mathbb{R}^1\}$). d and $|v|$ represent the dimension of token embeddings and the size of the token vocabulary, respectively. h_{cls} represents ("<CLS>") sequence embedding output from the final Transformer-encoder layer.

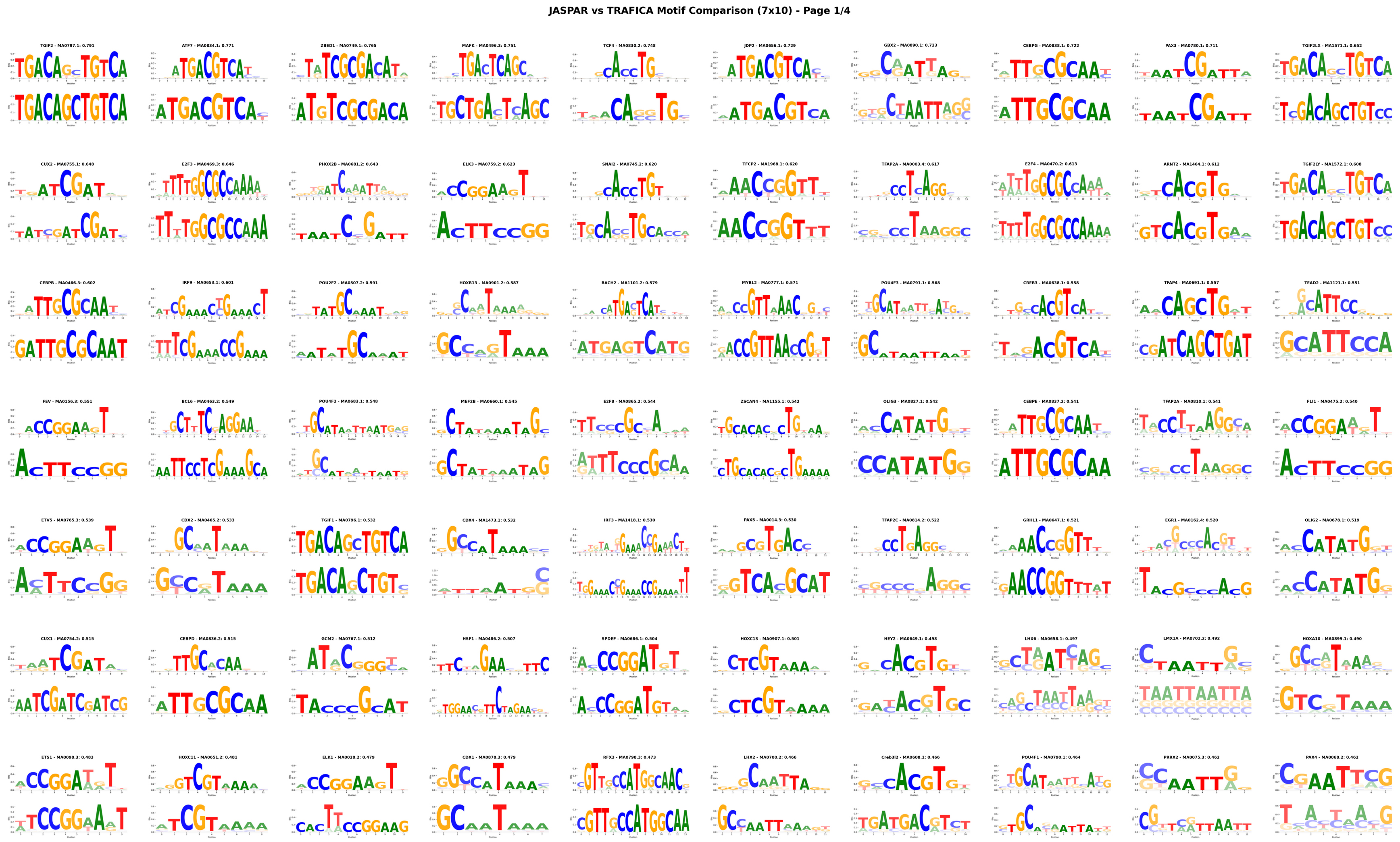

Figure S2. The logos of the motif pairs with the top-70 highest similarities. The top panel displays the validated TF binding motifs from the JASPAR database, whereas the bottom panel presents the TF binding motifs generated by the attention scores from TRAFICA. The titles of each subfigure indicate TF names, JASPAR ID, and similarity scores.

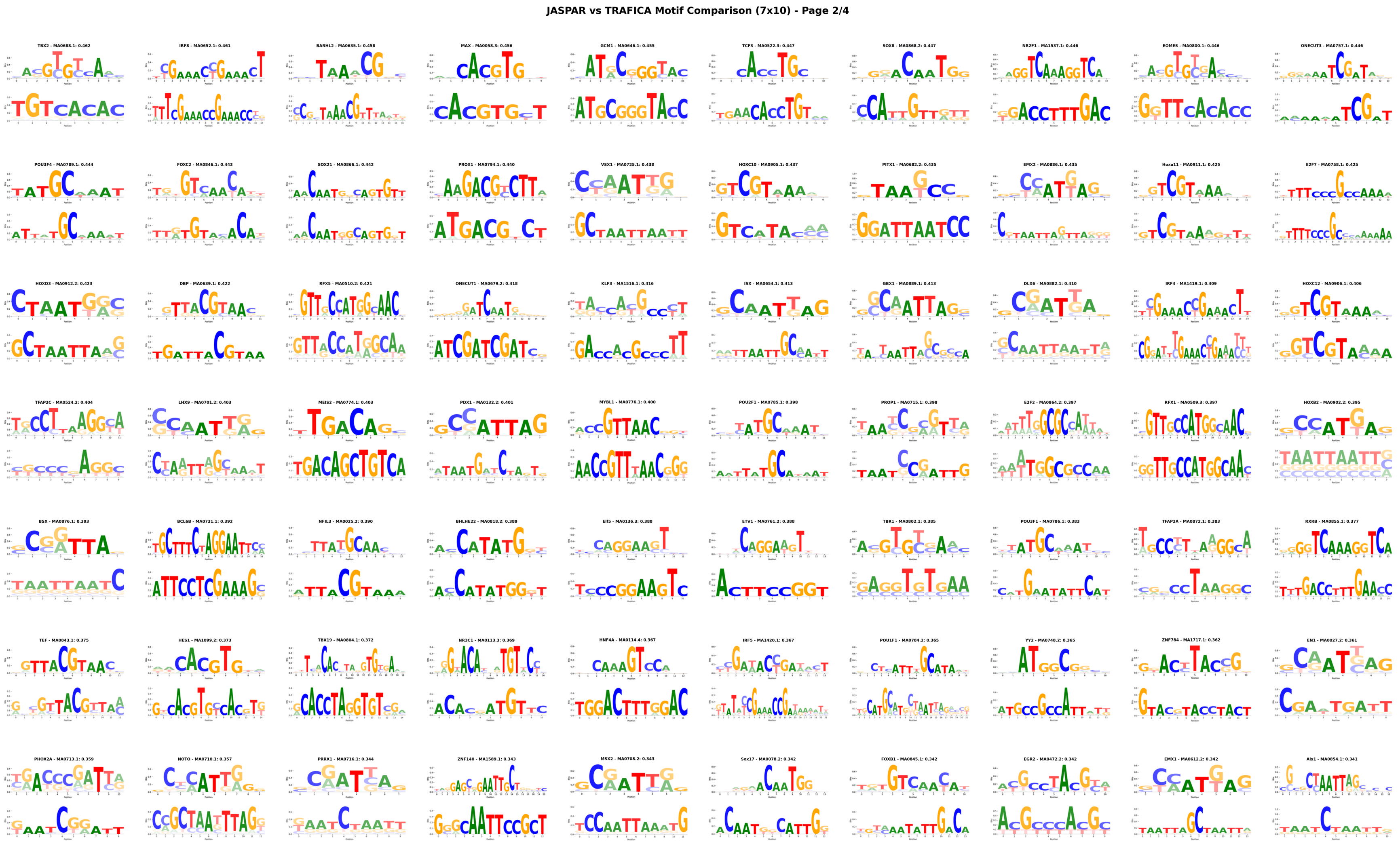

Figure S3. The logos of the motif pairs whose similarities ranked from 71 to 140.

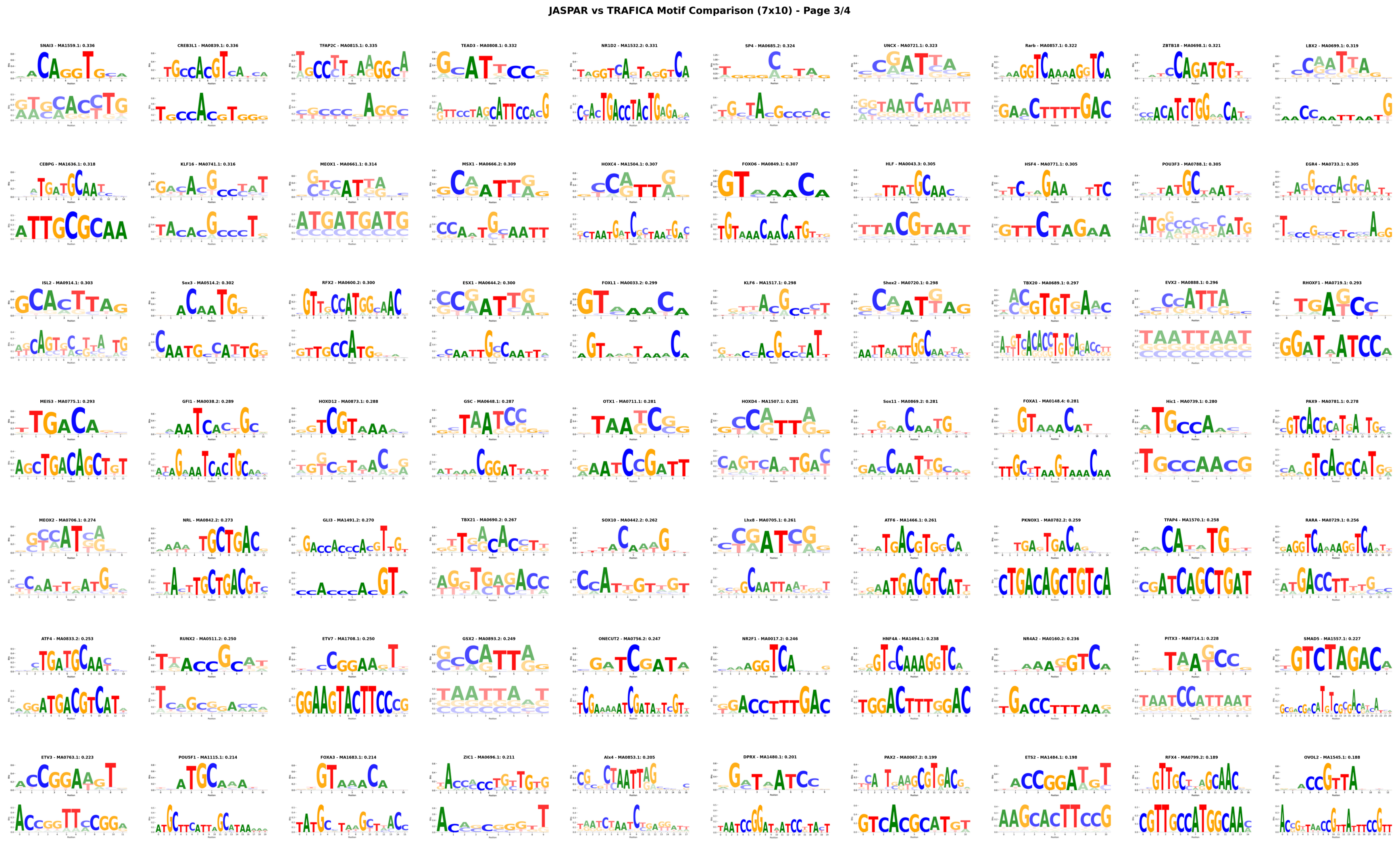

Figure S4. The logos of the motif pairs whose similarities ranked from 141 to 210.

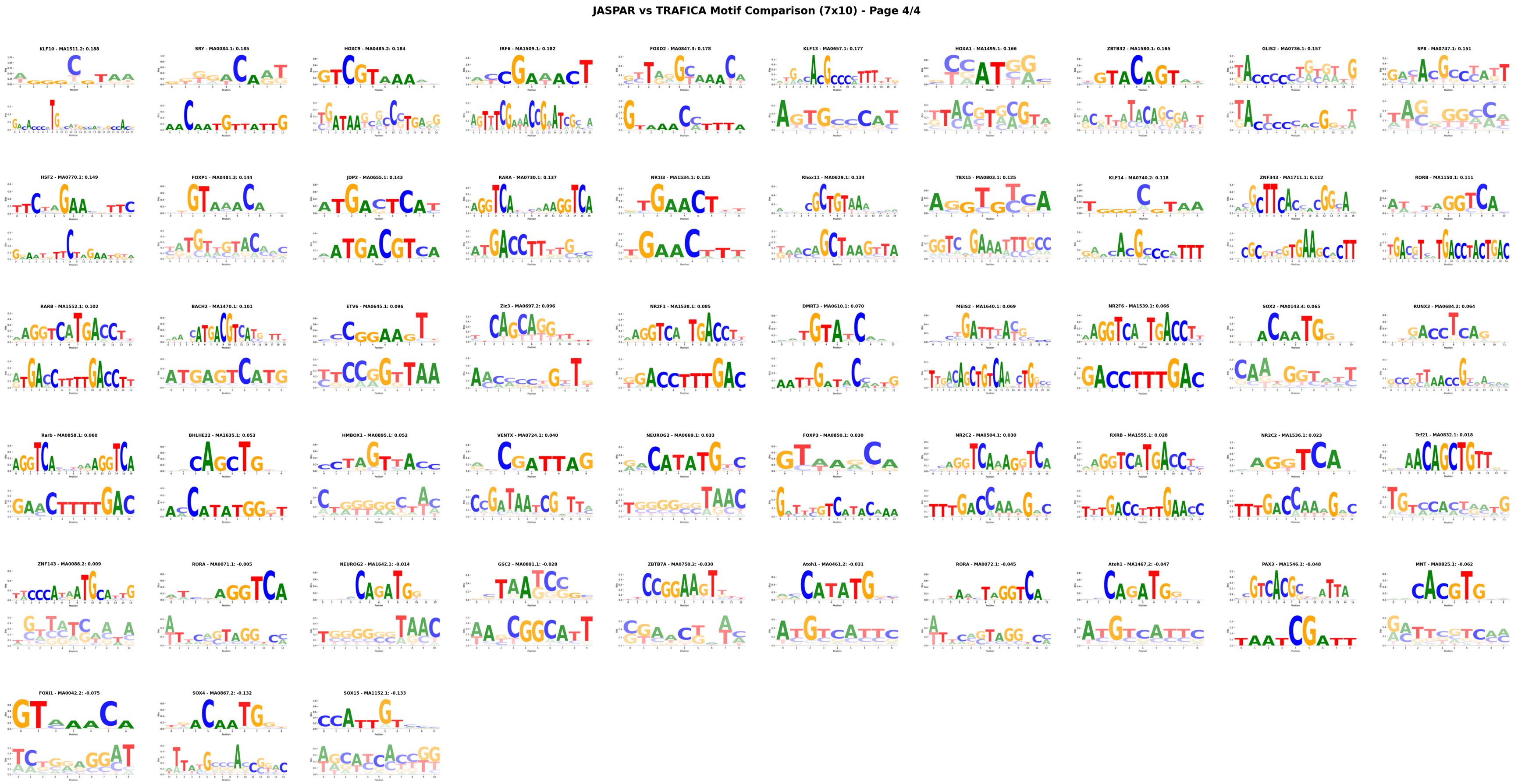

Figure S5. The logos of the motif pairs whose similarities ranked from 211 to 263.
